# Intermittent chemotherapy plus either intermittent or continuous cetuximab for first-line treatment of patients with *KRAS* wild-type advanced colorectal cancer (COIN-B): a randomised phase 2 trial

**DOI:** 10.1016/S1470-2045(14)70106-8

**Published:** 2014-05

**Authors:** Harpreet Wasan, Angela M Meade, Richard Adams, Richard Wilson, Cheryl Pugh, David Fisher, Benjamin Sydes, Ayman Madi, Bruce Sizer, Charles Lowdell, Gary Middleton, Rachel Butler, Richard Kaplan, Tim Maughan, on behalf of the COIN-B investigators

**Affiliations:** a Imperial College Healthcare NHS Trust, London, UK; b MRC Clinical Trials Unit at UCL, London, UK; c Cardiff University, Cardiff, UK; d Centre for Cancer Research and Cell Biology, Queen's University Belfast, Belfast, UK; e Essex County Hospital, Colchester, UK; f University of Birmingham, Birmingham, UK; g University Hospital of Wales, Cardiff, UK; h Cancer Research UK–MRC Gray Institute for Radiation Oncology and Biology, University of Oxford, Oxford, UK

## Abstract

**Background:**

Advanced colorectal cancer is treated with a combination of cytotoxic drugs and targeted treatments. However, how best to minimise the time spent taking cytotoxic drugs and whether molecular selection can refine this further is unknown. The primary aim of this study was to establish how cetuximab might be safely and effectively added to intermittent chemotherapy.

**Methods:**

COIN-B was an open-label, multicentre, randomised, exploratory phase 2 trial done at 30 hospitals in the UK and one in Cyprus. We enrolled patients with advanced colorectal cancer who had received no previous chemotherapy for metastases. Randomisation was done centrally (by telephone) by the Medical Research Council Clinical Trials Unit using minimisation with a random element. Treatment allocation was not masked. Patients were assigned (1:1) to intermittent chemotherapy plus intermittent cetuximab or to intermittent chemotherapy plus continuous cetuximab. Chemotherapy was FOLFOX (folinic acid and oxaliplatin followed by bolus and infused fluorouracil). Patients in both groups received FOLFOX and weekly cetuximab for 12 weeks, then either had a planned interruption (those taking intermittent cetuximab) or planned maintenance by continuing on weekly cetuximab (continuous cetuximab). On RECIST progression, FOLFOX plus cetuximab or FOLFOX was recommenced for 12 weeks followed by further interruption or maintenance cetuximab, respectively. The primary outcome was failure-free survival at 10 months. The primary analysis population consisted of patients who completed 12 weeks of treatment without progression, death, or leaving the trial. We tested *BRAF* and *NRAS* status retrospectively. The trial was registered, ISRCTN38375681.

**Findings:**

We registered 401 patients, 226 of whom were enrolled. Results for 169 with *KRAS* wild-type are reported here, 78 (46%) assigned to intermittent cetuximab and 91 (54%) to continuous cetuximab. 64 patients assigned to intermittent cetuximab and 66 of those assigned to continuous cetuximab were included in the primary analysis. 10-month failure-free survival was 50% (lower bound of 95% CI 39) in the intermittent group versus 52% (lower bound of 95% CI 41) in the continuous group; median failure-free survival was 12·2 months (95% CI 8·8–15·6) and 14·3 months (10·7–20·4), respectively. The most common grade 3–4 adverse events were skin rash (21 [27%] of 77 patients *vs* 20 [22%] of 92 patients), neutropenia (22 [29%] *vs* 30 [33%]), diarrhoea (14 [18%] *vs* 23 [25%]), and lethargy (20 [26%] *vs* 19 [21%]).

**Interpretation:**

Cetuximab was safely incorporated in two first-line intermittent chemotherapy strategies. Maintenance of biological monotherapy, with less cytotoxic chemotherapy within the first 6 months, in molecularly selected patients is promising and should be validated in phase 3 trials.

**Funding:**

UK Medical Research Council, Merck KGaA.

## Introduction

The discovery of predictive biomarkers for advanced colorectal cancer and the development of new targeted treatments has led to the combination of cytotoxic drugs with targeted treatments as the international standard of care. However, these combinations have failed to improve outcomes in several phase 3 trials.^1^, ^2^, ^3^, ^4^ Toxic effects caused by drug combinations have also confounded assessments of efficacy.^2^, ^3^

Intermittent treatment and maintenance biological treatment have been explored in several trials to address this shortcoming.^3^, ^4^, ^5^, ^6^, ^7^, ^8^, ^9^, ^10^, ^11^ Palliative treatment of cancer should address both quantity and quality of life. Minimising the time spent taking cytotoxic drugs and introducing chemotherapy-free intervals or complete treatment holidays (ie, planned interruptions) might help to meet both these goals. De-escalation of components of treatment for maintenance in patients who have not progressed is increasingly done in practice and a clinical benefit has been shown in a trial of capecitabine and bevacizumab maintenance treatment.^8^ However, the best strategy to use for different clinically or molecularly defined cohorts has yet to be established.

The COIN trial^1^, ^6^ was designed to assess whether intermittent chemotherapy was as effective as continuous chemotherapy and whether the addition of cetuximab to continuous chemotherapy was associated with additional benefit. In the COIN-B trial—done as an adjunct to COIN—we sought to establish how cetuximab might be safely and effectively added to intermittent chemotherapy.

## Methods

### Study design and participants

We did this open-label, multicentre, randomised, exploratory phase 2 trial at 30 hospitals in the UK and one in Cyprus. Eligibility criteria were age 18 years or older, colorectal adenocarcinoma, inoperable metastatic or locoregional measurable disease according to RECIST (version 1.1), no previous chemotherapy for metastases, WHO performance status 0–2, and good organ function (baseline requirements were: ≥1·5 × 10^9^ neutrophils per L, ≥100 × 10^9^ platelets per L, serum bilirubin ≤1·25 × upper limit of normal, serum aminotransferases ≤2·5 × upper limit of normal, alkaline phosphatase ≤5 × upper limit of normal, and estimated creatinine clearance or measured glomerular filtration rate ≥50 mL/min). All patients were eligible irrespective of their EGFR status; however, consent was obtained for tumour sample collection. Patients were excluded if they had had any previous cancer, uncontrolled medical comorbidity likely to interfere with COIN-B treatment or response assessment, or known brain metastases.

The trial was designed before *KRAS* mutations were identified as predictors of resistance to EGFR monoclonal antibody treatment.^12^ COIN-B was suspended in May, 2008, and on restarting (January 2009) it included prospective *KRAS* mutation analysis before randomisation. From January, 2009, only patients whose tumours were *KRAS* wild-type were eligible. While the trial was suspended, the *KRAS* status of enrolled patients was assessed.

The trial protocol is available online. All patients gave written informed consent. COIN-B was approved by the South West Research ethics committee and the Medicines and Healthcare Regulatory Agency in the UK and the national Bioethics and the Pharmaceutical Services of the Ministry of Health in Cyprus.

### Randomisation and masking

The MRC Clinical Trials Unit did the randomisation by telephone, using the method of minimisation with a random element. The minimisation factors were hospital, WHO performance status, previous adjuvant chemotherapy, liver metastases, and peritoneal metastases. Patients were randomly assigned (1:1) to intermittent chemotherapy plus intermittent cetuximab or intermittent chemotherapy plus continuous cetuximab. Treatment allocation was not masked.

### Procedures

For prospective *KRAS* screening, tumour samples were obtained from the hospital of diagnosis. RB pyrosequenced *KRAS* codons 12, 13, and 61 with DNA extracted from macrodissected formalin-fixed paraffin-embedded sections. RB established the mutational status of *BRAF* (codon 600) and *NRAS* (codons 12, 13, and 61) retrospectively for *KRAS* wild-type patients who consented to future bowel cancer research. The appendix shows details of the primers used. We also constructed tumour microarrays for immunohistochemical analysis of EGFR.

The chemotherapy backbone was the UK FOLFOX regimen, consisting of an introvenous infusion of 175 mg l-folinic acid given concurrently with 85 mg/m^2^ oxaliplatin over 2 h, followed by a 400 mg/m^2^ intravenous bolus of fluorouracil over 5 min followed by 2400 mg/m^2^ fluorouracil intravenous infusion over 46 h.^1^ Cetuximab was given in an initial intravenous dose of 400 mg/m^2^ (first dose and on reintroduction) and subsequently at 250 mg/m^2^ once a week. Each cycle of FOLFOX included two doses of cetuximab on days 1 and 8. When cetuximab was given in combination with chemotherapy (day 1), cetuximab was given first (see protocol for full details of the treatment regimens).

**Figure 1:**
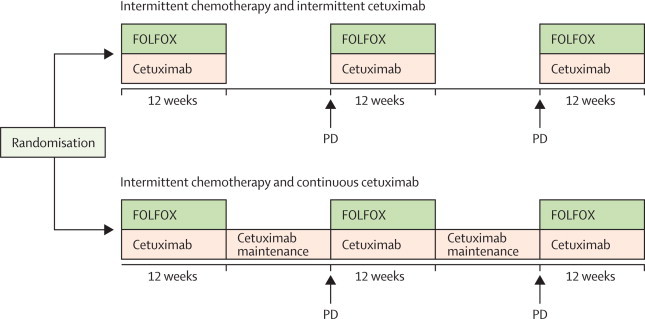
Trial design Treatment cycles continued until progressive disease (PD) with maximally tolerated treatment, or patient choice. FOLFOX=folinic acid and oxaliplatin followed by bolus and infused fluorouracil.

Patients in both groups received treatment for 12 weeks and those with stable or responding disease started a chemotherapy-free interval (ie, no FOLFOX). Patients assigned to the intermittent cetuximab group also ceased treatment with cetuximab, whereas patients assigned to continuous cetuximab had planned maintenance with continuous cetuximab monotherapy. In this period, patients had clinical assessments every 6 weeks and CT scans every 12 weeks. On RECIST confirmation of progressive disease, maximum tolerated treatment was restarted for a further 12 weeks (figure 1). The cycling of treatment and complete breaks or maintenance could be continued until progressive disease on maximally tolerated treatment or patient choice. The appendix provides details of permitted dose delays and modifications. If a patient had a grade 3 or 4 allergic reaction to cetuximab at any time cetuximab was discontinued.

Symptoms were scored with the National Cancer Institute Common Toxicity Criteria for Adverse Events (version 3.0). Serious adverse events and deaths—together with an assessment of causality—were continuously reported; and were reassessed by an experienced oncologist (first by AM, then the trial management group) on behalf of the Medical Research Council.

A baseline CT scan was done within 4 weeks before the start of treatment and then at least once every 12 weeks and evaluated with RECIST criteria. We did not confirm responses with repeat scans nor did we do central radiological review. Data were collected via remote data capture, except for reports of serious adverse events, which were submitted by fax.

### Outcomes

The primary outcome was failure-free survival at 10 months. A failure was defined as stopping maximum tolerated treatment as a result of progression or death (from any cause). The primary analysis population included patients who completed 12 weeks of COIN-B treatment (induction phase) without progression, death, or leaving the trial. Secondary objectives were: safety assessment of cetuximab reintroduction, overall survival, progression-free survival, response rates, toxic effects, disease control at 24 weeks (complete response, progressive disease, and stable disease), and quality of life.

Time from randomisation (week 0) was used for the analysis of failure-free survival and overall survival. Progression-free survival was analysed from 12 weeks (when the treatment plans of the two groups diverge). At the time of analysis, survivors were censored at the date they were last known to be alive.

The primary and main secondary outcomes (overall survival and progression-free survival in the interval) were retrospectively analysed for patients who were triple wild-type (for *KRAS, BRAF*, and *NRAS*). We also analysed biomarker status as a potential prognostic factor for failure-free survival and overall survival.

### Statistical analysis

In the MRC FOCUS trial,^13^ failure-free survival at 9 months was roughly 50% for patients treated with continuous oxaliplatin and infused fluorouracil chemotherapy, while median overall survival was 15·4 months with FOLFOX. Data from phase 2 studies^14^ suggested that 50% failure-free survival at 10 months would be a suitable primary outcome for the addition of cetuximab.

We designed COIN-B as two separately powered phase 2 trials, using A'Hern's single-stage design^15^ to distinguish between a 10-month failure-free survival of 50% (implying that the treatment would be worth pursuing in a phase 3 trial, feasibility and toxic effects permitting) and of 35% (implying that the treatment would not be worth pursuing). The trial was not powered for comparison between the treatment groups. The original design aimed to recruit 136 patients (irrespective of *KRAS* status, 68 per group) with a one-sided α of 5% and 80% power. During the trial's suspension, interim data from COIN and COIN-B suggested that attrition before 12 weeks was 16% because of toxic effects or absence of benefit. As a result, we changed the target enrolment so that 158 patients with *KRAS* wild-type would be included, of whom we expected 136 to be assessable for the primary outcome.

We used the Kaplan-Meier method to assess failure-free survival, overall survival, and progression-free survival. We used Stata (version 11.1) for all statistical analyses.

The trial is registered as an International Standard Randomised Controlled Trial, number ISRCTN38375681.

### Role of the funding source

The MRC was the overall sponsor of the trial. Staff from the MRC Clinical Trials Unit (who are employees of the MRC) were involved in the trial design and were responsible for all data collection, management, and analysis. Merck KGaA reviewed the report. The writing of the report and the decision to submit for publication was the responsibility of the COIN-B trial management group. The corresponding author had full access to the data and had final responsibility for the decision to submit for publication.

## Results

**Figure 2:**
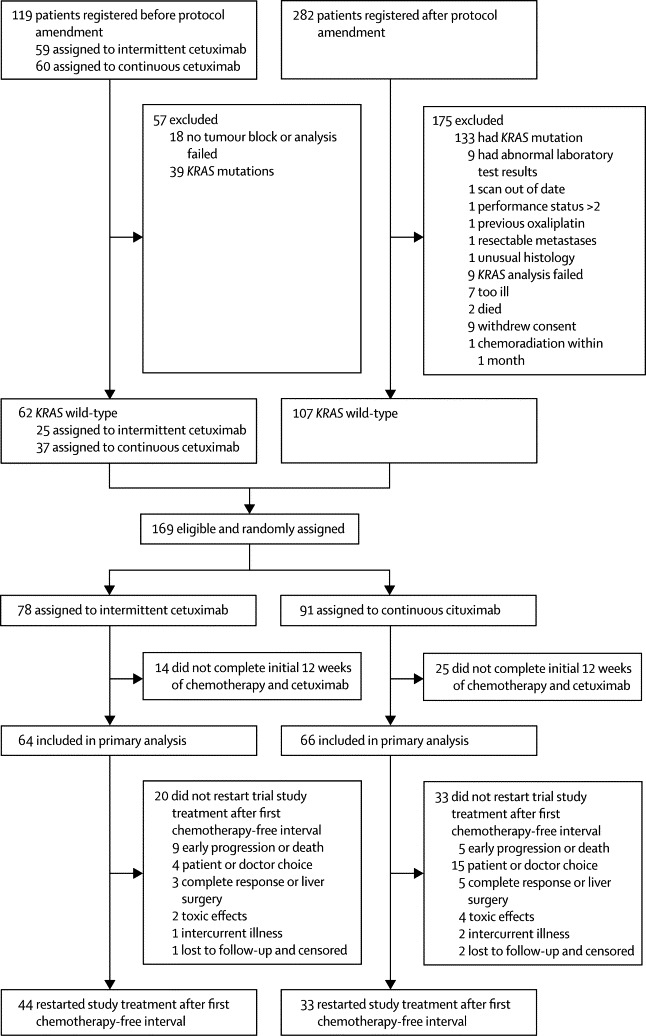
Trial profile

Between July 13, 2007, and March 6, 2010, 401 patients were registered and 226 patients were enrolled. 169 were *KRAS* wild-type. 78 *KRAS* wild-type patients were assigned to the intermittent cetuximab group and 91 to the continuous cetuximab group. More patients in the continuous cetuximab group were *KRAS* wild-type when tested retrospectively (figure 2). The two groups had some differences in baseline characteristics associated with poor prognosis (table 1). The continuous cetuximab group had more elderly patients (age >75 years), more with a WHO performance score of 2, more with *BRAF* mutations, and more with primary colon cancers (*vs* rectal) than did the intermittent cetuximab group (Table 1, Table 2).

**Table 1:** Baseline characteristics

		***KRAS* wild-type patients**		**All randomised patients**	
		Intermittent cetuximab (n=78)	Continuous cetuximab (n=91)	Intermittent cetuximab (n=112)	Continuous cetuximab (n=114)
Sex					
	Men	48 (62%)	55 (60%)	66 (59%)	65 (57%)
	Women	30 (38%)	36 (40%)	46 (41%)	49 (43%)
Median age at randomisation (IQR; years)		63 (56–70)	64 (54–71)	64 (57–70)	65 (56–71)
Age >75 years		5 (6%)	12 (13%)	9 (8%)	15 (13%)
WHO performance status					
	0	38 (49%)	40 (44%)	54 (48%)	53 (46%)
	1	35 (45%)	42 (46%)	50 (45%)	51 (45%)
	2	5 (6%)	9 (10%)	8 (7%)	10 (9%)
Site of primary tumour					
	Right or transverse colon	23 (29%)	29 (32%)	32 (29%)	37 (32%)
	Left colon or RSJ	30 (38%)	45 (49%)	40 (36%)	56 (49%)
	Rectum	25 (32%)	17 (19%)	40 (36%)	21 (18%)
Status of primary tumour					
	Resected	41 (53%)	48 (53%)	58 (52%)	57 (50%)
	Unresected	34 (44%)	41 (45%)	50 (45%)	55 (48%)
	Local recurrence	3 (4%)	2 (2%)	4 (4%)	2 (2%)
Distribution of metastases					
	Liver only	16 (21%)	17 (19%)	21 (19%)	24 (21%)
	Liver and elsewhere	39 (50%)	43 (47%)	58 (52%)	55 (48%)
	Non-liver	22 (28%)	30 (33%)	32 (29%)	34 (30%)
	Data missing[A: Ok?]	1 (1%)	1 (1%)	1 (1%)	1 (1%)
Number of metastatic sites					
	None	1 (1%)	1 (1%)	1 (1%)	1 (1%)
	One	28 (36%)	31 (34%)	39 (35%)	41 (36%)
	Two	22 (28%)	35 (38%)	37 (33%)	47 (41%)
	Three	27 (35%)	24 (26%)	35 (31%)	25 (22%)
Adjuvant chemotherapy					
	No	62 (79%)	73 (80%)	90 (80%)	92 (81%)
	1–6 months ago	4 (5%)	2 (2%)	5 (4%)	4 (4%)
	>6 months ago	12 (15%)	16 (18%)	17 (15%)	18 (16%)

Data are n (%) unless stated otherwise. RSJ= rectosigmoid junction.

**Table 2:** Genetics of participants

	**Intermittent cetuximab (n=78)**	**Continuous cetuximab (n=91)**
All wild-type	53 (68%)	58 (64%)
*NRAS* mutation	7 (9%)	8 (9%)
*BRAF* mutation	8 (10%)	16 (18%)
*NRAS* inconclusive, *BRAF* wild-type	1 (1%)	1 (1%)
*NRAS* wild-type, *BRAF* inconclusive	0 (0%)	1 (1%)
Both inconclusive	0 (0%)	1 (1%)
Mutation analysis not possible*	9 (12%)	6 (6%)

Data are n (%). *KRAS* wild-type population only.

* Lack of appropriate consent or lack of sample.

24 patients had *BRAF* mutations and 15 had *NRAS* mutations (table 2). 111 (66%) of 169 patients had wild-type *KRAS, BRAF*, and *NRAS* (table 2). When the trial started, *EGFR* expression was the most plausible biomarker for cetuximab-related benefit. It has not since been validated and our analysis confirms that it is not informative (data not shown).

At the time of analysis (April 24, 2012), median duration of follow-up in the *KRAS* wild-type intention-to-treat population was 32·8 months (IQR 22·9–45·8) in the intermittent cetuximab group and 34·2 months (IQR 27·3–50·4 months) in continuous cetuximab group.

Seven patients did not start protocol treatment: two in the intermittent cetuximab group and five in the continuous cetuximab group. Two patients were found to be ineligible after randomisation (one in each group) and five patients died before treatment was initiated (one in the intermittent cetuximab group *vs* four in the continuous cetuximab group). 13 patients (five *vs* eight) received capecitabine during treatment. Total dose of trial drug and dose intensity within the first 12 weeks was much the same in each group (data not shown). Most patients required a treatment delay, 68 (87%) of 78 patients in the intermittent cetuximab group and 74 (81%) of 91 in the continuous cetuximab group. Dose modifications occurred in similar proportions in each group; for cetuximab, modifications were made for 57 (73%) of 78 patients taking intermittent cetuximab versus 70 (77%) of 91 taking continuous cetuximab, oxaliplatin was modified for 40 (51%) versus 43 (47%) patients, and fluorouracil was modified for 49 (63%) versus 51 (56%). Treatment was discontinued because of drug-related toxic effects for nine (12%) of 78 patients in the intermittent group versus 11 (12%) of 91 in the continuous group.

**Figure 3:**
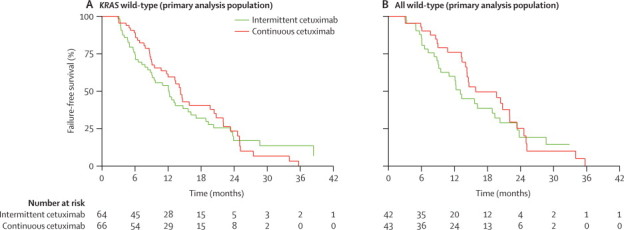
Kaplan-Meier analyses of failure-free survival

The primary analysis included 64 patients in the intermittent cetuximab group and 66 in the continuous cetuximab group. Patients were excluded for progressive disease (one in the intermittent cetuximab group *vs* four in the continuous cetuximab group), death (four *vs* 14), and failure of treatment in the first 12 weeks (nine *vs* seven). The greater dropout from the continuous cetuximab group could be a result of differences in baseline characteristics combined with the greater proportion of patients with a *BRAF* mutation (table 1, appendix). In the primary analysis population, at least 50% of patients were failure-free at 10 months in both groups: 32 (50%) of 64 in the intermittent cetuximab group and 34 (52%) of 66 in the continuous cetuximab group. The lower bound of the 95% CI was 39% and 41%, respectively; therefore both groups also exceeded the pre-defined 35% futility limit. Median failure-free survival was 12·2 months (95% CI 8·8–15·6) in the intermittent cetuximab group and 14·3 months (10·7–20·4) in the continuous cetuximab group (figure 3A). In an intention-to-treat analysis (n=169), median failure-free survival was 12·1 months (95% CI 7·8–14·7) versus 12·0 months (8·7–14·5; appendix). Median failure-free survival was greater for patients with all wild-type alleles than for those with *KRAS* wild-type only in both treatment groups, in both the primary analysis cohort and the intention-to-treat population (figure 3B; appendix).

**Figure 4:**
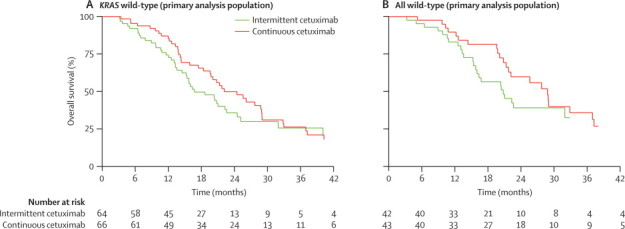
Kaplan-Meier analyses of overall survival

In the primary analysis cohort, median overall survival was 16·8 months (95% CI 14·5–22·6) in the intermittent cetuximab group versus 22·2 months (18·4–28·9) in the continuous cetuximab group (figure 4A). The difference in median overall survival was smaller in the intention-to-treat population (16·0 months, 95% CI 13·3–20·4 *vs* 17·5 months, 13·7–21·7; appendix). Median overall survival was greater for patients with all wild-type alleles than for those with *KRAS* wild-type only in both treatment groups in both the primary analysis cohort and the intention-to-treat population (figure 4B; appendix).

**Figure 5:**
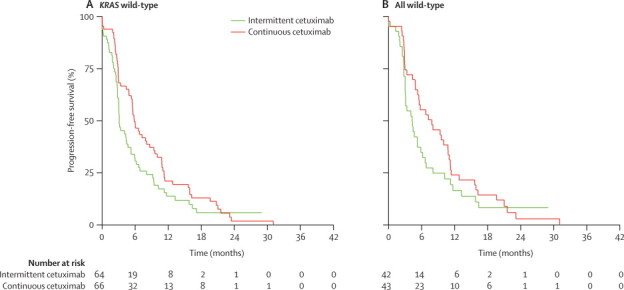
Kaplan-Meier analyses of progression-free survival during the chemotherapy-free interval

In the primary analysis cohort, median progression-free survival from week 12 was 3·1 months (95% CI 2·8–4·7) in the intermittent cetuximab group and 5·8 months (4·9–8·6) with continuous cetuximab (figure 5A). As with the other survival endpoints, median progression-free survival was greater for patients with all wild-type alleles than for those with *KRAS* wild-type, for both treatment groups (figure 5B).

54 (32%) of 78 patients died in the intermittent cetuximab group versus 67 (40%) of 91 in the continuous cetuximab group. 112 (93%) deaths were caused by colorectal cancer (49 *vs* 63), three (2%) were related to treatment (one *vs* two), and six (5%) were the result of other causes (four *vs* two).

Only 77 patients restarted trial treatment after a chemotherapy-free interval, 44 taking intermittent cetuximab and 33 taking continuous cetuximab. Median chemotherapy-free interval was 3·7 months (95% CI 3·5–4·6) versus 5·5 months (3·4–7·5; p=0·042; figure 2, appendix).

Patients in both groups received the same treatment for the first 12 weeks, yet a greater proportion of patients in the intermittent cetuximab group had a complete or partial response (49 of 78 [63%, 95% CI 51–74]) than did those receiving continuous cetuximab (39 of 91 [43%, 95% CI 33–54]). This finding is probably a result of imbalances in patients' baseline characteristics. For patients who survived at least 24 weeks, greater disease control (ie, complete response, partial response, or stable disease) was noted in those assigned to continuous cetuximab (n=29, 32%) than in those assigned to intermittent cetuximab (n=17, 22%), despite the imbalances.

Patients with mutations in *KRAS, NRAS*, and *BRAF* had a worse prognosis than patients with wild-type for overall survival and failure-free survival in the intention-to-treat population (p<0·0001 for both outcomes; appendix).

Toxic effects and adverse events were similar in each treatment group. The most common grade 3–4 adverse events were skin rash (21 [27%] of 77 in the intermittent cetuximab group *vs* 20 [22%] of 92 in the continuous cetuximab group, neutropenia (22 [29%] *vs* 30 [33%]), diarrhoea (14 [18%] *vs* 23 [25%]), and lethargy (20 [26%] *vs* 19 [21%]). Three patients in the intermittent cetuximab group reported grade 3 or higher hypersensitivity events with only one on reintroduction of cetuximab (table 3).

**Table 3:** Toxic effects

	**Intermittent cetuximab (n=78)**					**Continuous cetuximab (n=91)**				
	Grade 0	Grade 1	Grade 2	Grade 3	Grade 4	Grade 0	Grade 1	Grade 2	Grade 3	Grade 4
Nausea	17 (22%)	39 (50%)	16 (21%)	6 (8%)	0 (0%)	37 (41%)	32 (35%)	18 (20%)	4 (4%)	0 (0%)
Vomiting	37 (47%)	19 (24%)	13 (17%)	8 (10%)	1 (1%)	58 (64%)	1618%)	12 (13%)	5 (5%)	0 (0%)
Anorexia	22 (28%)	24 (31%)	26 (33%)	6 (8%)	0 (0%)	31 (34%)	32 (35%)	25 (27%)	3 (3%)	0 (0%)
Pain	13 (17%)	29 (37%)	21 (27%)	12 (15%)	3 (4%)	21 (23%)	21 (23%)	32 (35%)	16 (18%)	1 (1%)
Stomatitis	14 (18%)	26 (33%)	29 (37%)	9 (12%)	0 (0%)	22 (24%)	28 (31%)	35 (38%)	6 (7%)	0 (0%)
Diarrhoea	16 (21%)	24 (31%)	24 (31%)	14 (18%)	0 (0%)	17 (19%)	32 (35%)	19 (21%)	22 (24%)	1 (1%)
Lethargy	7 (9%)	20 (26%)	31 (40%)	18 (23%)	2 (3%)	15 (16%)	24 (26%)	33 (36%)	18 (20%)	1 (1%)
Thrombocytopenia	37 (47%)	35 (45%)	4 (5%)	2 (3%)	0 (0%)	49 (54%)	28 (31%)	11 (12%)	2 (2%)	1 (1%)
Abnormal haemoglobin concentration	17 (22%)	28 (36%)	28 (36%)	3 (4%)	2 (3%)	28 (31%)	36 (40%)	23 (25%)	3 (3%)	1 (1%)
Leucopenia	37 (47%)	17 (22%)	15 (19%)	8 (10%)	1 (1%)	45 (49%)	16 (18%)	18 (20%)	11 (12%)	1 (1%)
Neutropenia	32 (41%)	13 (17%)	11 (14%)	16 (21%)	6 (8%)	40 (44%)	10 (11%)	11 (12%)	22 (24%)	8 (9%)
Skin rash	6 (8%)	18 (23%)	33 (42%)	21 (27%)	0 (0%)	12 (13%)	18 (20%)	41 (45%)	20 (22%)	0 (0%)
Hand-foot syndrome	26 (33%)	25 (32%)	21 (27%)	6 (8%)	0 (0%)	30 (33%)	21 (23%)	33 (36%)	7 (8%)	0 (0%)
Peripheral neurotoxicity	12 (15%)	41 (53%)	21 (27%)	4 (5%)	0 (0%)	18 (20%)	52 (57%)	17 (19%)	4 (4%)	0 (0%)
Hypomagnesaemia	44 (56%)	25 (32%)	6 (8%)	1 (1%)	2 (3%)	44 (48%)	35 (38%)	6 (7%)	4 (4%)	2 (2%)
Cetuximab hypersensitivity	70 (90%)	4 (5%)	1 (1%)	3 (4%)	0 (0%)	76 (84%)	9 (10%)	6 (7%)	0 (0%)	0 (0%)

Data are n (%). For *KRAS* wild-type patients.

Both the COIN and COIN-B trials contained a quality-of-life substudy. Patient and carer participation was optional and compliance in COIN-B was low, with 42 patients and 23 carers consenting to participate. Results from these substudies will be presented separately.

## Discussion

Our findings show that cetuximab can be safely and effectively incorporated in two intermittent chemotherapy strategies, where the cytotoxic doublet induction chemotherapy is given for only 3 months. Although not statistically compared, maintenance cetuximab with intermittent chemotherapy seemed to be slightly more active than intermittent cetuximab with intermittent chemotherapy.

COIN-B is one of a series of trials of patients with non-curable advanced colorectal cancer to explore strategies to improve the combinations and sequences of cytotoxic chemotherapy, newer targeted treatments, planned maintenance, and planned interruptions; all with a focus on reducing toxic effects and improving quality of life without reducing survival. The emergence of clinical and molecular biomarkers—both prognostic and predictive—has enabled these strategies to be further refined. In some trials, part or all of the first-line chemotherapy is discontinued in the investigational group and compared with continuation of the full first-line treatment.^4^, ^7^, ^11^, ^16^ In others, planned maintenance is compared with a planned interruption^5^, ^8^, ^9^ or an additional maintenance treatment is introduced when chemotherapy is discontinued.^10^ In all these studies, the conventional surrogate endpoint of progression-free survival is inappropriate because it does not consider the subsequent benefit of planned reintroduction of full first-line treatment on progression. Other outcome measures have therefore been used (duration of disease control and failure-free survival).^5^ However, the best measure of how maintenance treatment affects the course of disease is progression-free survival in the interval.

The COIN trial adds to preliminary data from CR06 and OPTIMOX-1,^11^, ^16^ which suggested that interruption or de-escalation of treatment was safe and potentially beneficial. The results of Adams and colleagues^6^ could not exclude the possibility of a very small negative effect on overall survival of a treatment holiday after 3 months of cytotoxic chemotherapy. The upper limits of the two-sided 80% CIs for the hazard ratios (HRs) in both the per-protocol and intention-to-treat analyses were greater than the predefined non-inferiority boundary of 1·162. The HR in the intention-to-treat population (n=1630) was 1·084 (80% CI 1·008–1·165) and in the per-protocol population (n=978) it was 1·087 (0·986–1·198).^6^ Planned subgroup analyses showed that patients with normal baseline platelet counts could gain the benefits of intermittent chemotherapy without harming survival. This finding suggests that a planned interruption might not be detrimental to most patients and warrants further study with clinical or molecular predictive markers.

COIN-B was designed as an exploratory, hypothesis-generating study to complement COIN. At randomisation, only infusional fluorouracil in combination with oxaliplatin and cetuximab was allowed, which removed the confounding effect and possible negative interaction reported in COIN and other studies when capecitabine was the partner fluoropyrimidine.^17^ In COIN-B, planned maintenance with cetuximab (ie, continuous cetuximab) was associated with a greater failure-free survival, greater progression-free survival, greater overall survival, improved disease control at 24 weeks, and a longer chemotherapy-free interval than was intermittent cetuximab. These benefits occurred despite an imbalance of prognostic factors at baseline. Such an imbalance might be considered a limitation of a small study such as COIN-B; however, the main reason for the imbalance between groups was discovered after the change in the population of interest from all patients with advanced colorectal cancer to those who had *KRAS* wild-type tumours. 119 patients were enrolled before the introduction of *KRAS* screening, 62 of whom were subsequently found to be *KRAS* wild-type and included in the primary outcome analysis. Of these 62, 25 had been previously assigned to the intermittent group and 37 had been assigned to continuous cetuximab. When prospective *KRAS* screening was introduced, 107 patients were randomly assigned: 52 to intermittent cetuximab and 55 to continuous cetuximab. Furthermore, the benefits of planned maintenance were even greater in patients who were triple wild-type (*KRAS, NRAS*, and *BRAF*). This finding is consistent with data for EGFR inhibitors as first-line treatment.^18^, ^19^

Other studies comparing planned maintenance treatment with planned interruption have shown evidence of a benefit of maintenance treatment. In OPTIMOX-2, planned maintenance with infusions of fluorouracil and leucovorin prolonged disease control compared with a planned interruption although it had no significant effect on overall survival (23·8 months *vs* 19·5 months, HR 0·85; p=0·42).^5^ In CAIRO-3,^8^ planned maintenance with bevacizumab and capecitabine was compared with a planned interruption, after 4 months of induction treatment with capecitabine, oxaliplatin, and bevacizumab. Planned maintenance improved progression-free survival in the interval (4·1 months *vs* 8·5 months, HR 0·44, 95% CI 0·37–0·54; p<0·0001) and overall survival (17·9 months *vs* 21·7 months, HR 0·77, 95% CI 0·62–0·96; p=0·02). In the SAKK study,^9^ non-inferiority of a planned interruption of all treatment compared with maintenance bevacizumab could not be shown. Median time to progression was 17·9 weeks (95% CI 13·3–23·4) for bevacizumab continuation and 12·6 weeks (12·0–16·4) for no continuation (HR 0·72, 95% CI 0·56–0·92). COIN-B is the first trial to enrol patients with *KRAS* wild-type to different intermittent treatment schedules and therefore show the effect of planned maintentance with a targeted monotherapy in a molecularly selected subgroup.

Two other studies have tested *EGFR* inhibition as planned maintenance treatment. The DREAM study^10^ compared bevacizumab alone or combined with erlotinib after 4–6 months of induction with bevacizumab-based chemotherapy. Progression-free survival in the interval was improved from 4·8 months with bevacizumab alone to 5·9 months with bevacizumab and erlotinib (HR 0·76, 95% CI 0·61–0·94; p=0·01). No difference in overall survival was recorded. No molecular selection criteria were applied to the randomly assigned cohort, which remains a serious limitation to bevacizumab monotherapy as a maintenance strategy for colorectal cancer. Thus, the best strategies by which to integrate bevacizumab and cetuximab into treatment are different, because no predictive biomarker has been discovered for bevacizumab.

The NORDIC-VII trial^4^ investigated the efficacy of cetuximab when added to bolus fluorouracil with folinic acid and oxaliplatin (Nordic FLOX) in patients with previously untreated metastatic colorectal cancer. Patients were randomly assigned to receive standard Nordic FLOX, cetuximab and Nordic FLOX, or cetuximab combined with intermittent Nordic FLOX. *KRAS* status was assessed retrospectively. Overall survival was almost identical in all three groups (20·4 months *vs* 19·7 months *vs* 20·3 months). The investigators did not compare planned maintenance with cetuximab with a planned interruption and had no mandated criteria for chemotherapy reintroduction; however, the similarity in overall survival between groups suggests that maintenance cetuximab might be an alternative strategy to continuing chemotherapy until progression.

The life expectancy of patients with advanced colorectal cancer is increasing, raising questions of duration and intensity of treatment required. Initial induction treatment followed by de-escalation of cytotoxic drugs and planned maintenance treatment is gaining credence, similar to treatment strategies for acute myeloid leukaemia. For EGFR inhibitors, greater refinement of biomarker selection suggests maintenance monotherapy might be a favourable continuation strategy (panel).

Panel **Research in context**

**Systematic review**

At the time of study inception, very few randomised trials had been done of cetuximab and chemotherapy, all of which were reviewed to help power the study, and choose the outcome measures. Stopping and restarting cetuximab could in theory increase the risk of hypersensitivity, so establishing safety was a main aim. We designed COIN-B to complement the COIN trial.^1^, ^6^ COIN first tested the non-inferiority of intermittent chemotherapy compared with continuous chemotherapy and second, the addition of cetuximab to continuous chemotherapy for superiority. No systematic review of the scientific literature was done.

**Interpretation**

In this trial, cetuximab was safely incorporated in two novel intermittent chemotherapy strategies. Continuous cetuximab (as planned maintenance monotherapy) was associated with a higher failure-free survival, longer chemotherapy-free interval, and longer time to progression. This finding needs to be validated in phase 3 trials, such as the upcoming FOCUS 4 study,^20^ in which novel targeted treatments will be assessed in biomarker-enriched populations.

The molecular evolution of panitumumab resistance has been elegantly shown in patients who were initially diagnosed as *KRAS* wild-type but soon developed detectable mutations in *KRAS* in their sera (three of whom developed several different *KRAS* mutations).^21^ The appearance of these mutations consistently occurred 5–6 months after the start of treatment, which coincides with the clinical benefit noted with *EGFR* inhibitors used as monotherapy. This could explain why solid tumours develop resistance to targeted treatments in a highly stereotypical fashion. The hypothesis that minimal residual disease in cancer is controlled, but rarely eradicated, implies that on–off strategies with drugs might be a rational way to regulate clonal selection favourably in palliative care. Our findings show that targeted treatment in a molecularly selected subgroup seems to improve outcomes and might delay the onset of clinical resistance. Other studies concur with COIN-B with respect to the benefit of planned maintenance, although both the best duration of induction chemotherapy and the benefit of continuing maintenance cytotoxic drugs to achieve maximal clinical benefit, are unknown. Further clinical trials are warranted to investigate these points.

For the **study protocol** see http://www.ctu.mrc.ac.uk/plugins/StudyDisplay/protocols/COIN-B%20Combined%20Protocol%20with%20CRFs.pdf
