# Supplementary material for: Intermittent chemotherapy plus either intermittent or continuous cetuximab for first-line treatment of patients with KRAS wild-type advanced colorectal cancer (COIN-B): a randomised phase 2 trial
Source: Lancet Oncol. 2014 May;15(6):631–9. doi: 10.1016/S1470-2045(14)70106-8 (PMC4012566; doi:10.1016/S1470-2045(14)70106-8)
Supplement: Supplementary appendix [file mmc1.pdf]

THE LANCET Oncology

Supplementary appendix

This appendix formed part of the original submission and has been peer reviewed. We post it as supplied by the authors.

Supplement to: Wasan H, Meade AM, Adams R, et al, on behalf of the COIN-B investigators. Intermittent chemotherapy plus either intermittent or continuous cetuximab for first-line treatment of patients with KRAS wild-type advanced colorectal cancer (COIN-B): a randomised phase 2 trial. *Lancet Oncol* 2014; published online April 3. http://dx.doi.org/10.1016/S1470-2045(14)70106-8.

COIN-B Supplementary Appendix

Supplementary Figures and Tables

Figure 1: Treatment breaks diagram
Figure 2: Analysis of Failure-Free Survival, ITT population
Figure 3: Analysis of Overall Survival, ITT population
Figure 4: Prognostic effects of mutational status, ITT population

Table 1: Description of Patients who Dropped out of Trial Therapy in First 12 weeks

Figure 1: Treatment Breaks Diagram

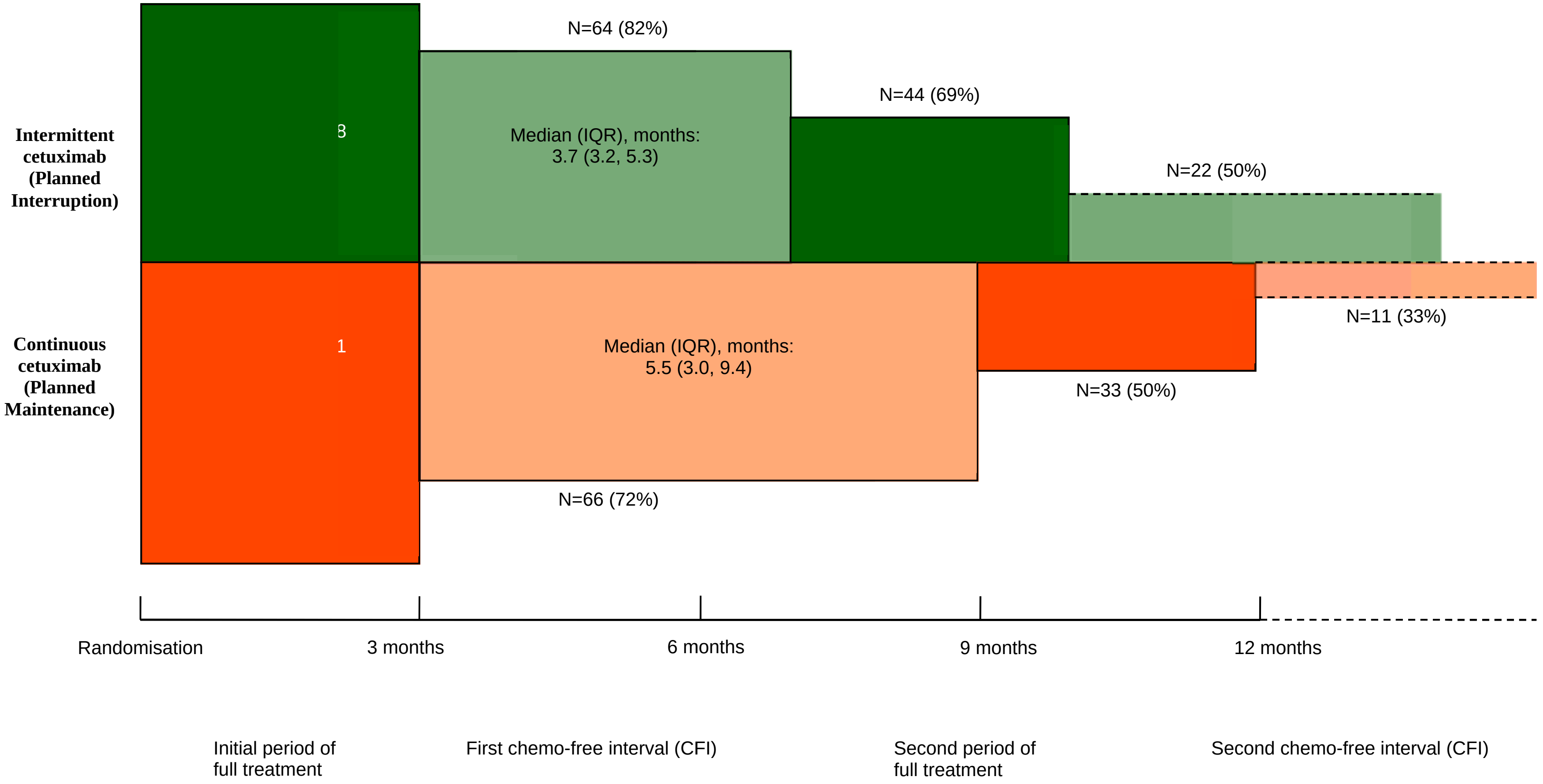

Figure 2: Kaplan-Meier Analyses of Failure-Free Survival, ITT population

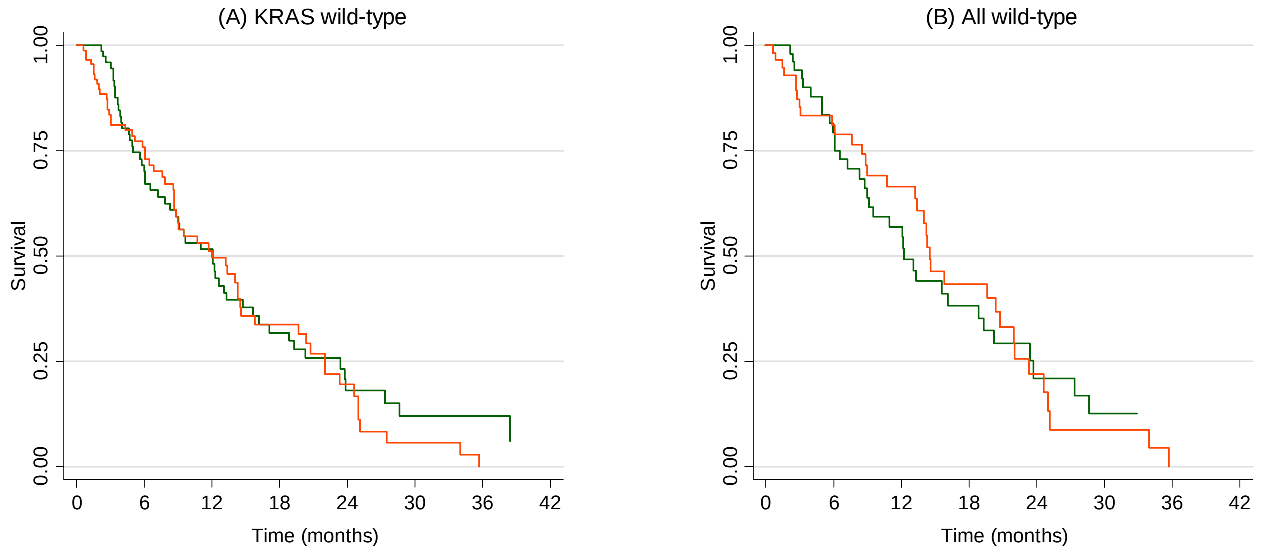

(A) KRAS wild-type – Number at risk

Number at risk	0	6	12	18	24	30	36	42
ICT+IC	78	47	30	16	6	3	2	1
ICT+CC	91	55	29	15	8	2	0	0

(B) All wild-type – Number at risk

Number at risk	0	6	12	18	24	30	36	42
ICT+IC	53	37	22	13	5	2	1	1
ICT+CC	58	36	24	13	6	2	0	0

ICT+IC ICT+CC

Median (95% CI) FFS (months):
KRAS wild-type: Intermittent cetuximab 12.1 (7.8, 14.7); Continuous cetuximab 12.0 (8.7, 14.5)
All wild-type: Intermittent cetuximab 12.3 (9.0, 18.9); Continuous cetuximab 14.5 (13.2, 20.7)

Figure 3: Kaplan-Meier Analyses of Overall Survival, ITT population

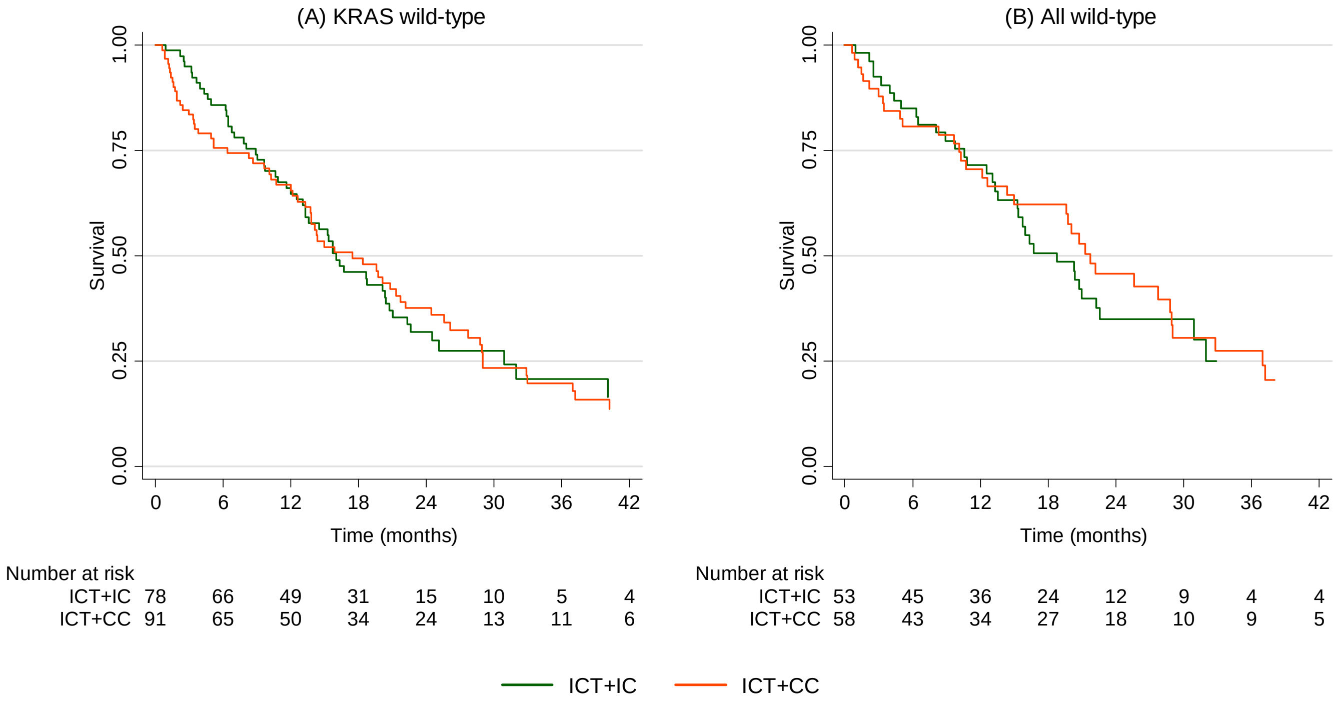

Median (95% CI) OS (months):
KRAS wild-type: Intermittent cetuximab 16.0 (13.3, 20.4); continuous cetuximab 17.5 (13.7, 21.7)
All wild-type: Intermittent cetuximab 18.8 (13.6, 22.6); continuous cetuximab 21.7 (14.4, 28.9)

Figure 4: Kaplan-Meier Analyses of Prognostic Effects of Mutational Status, ITT population

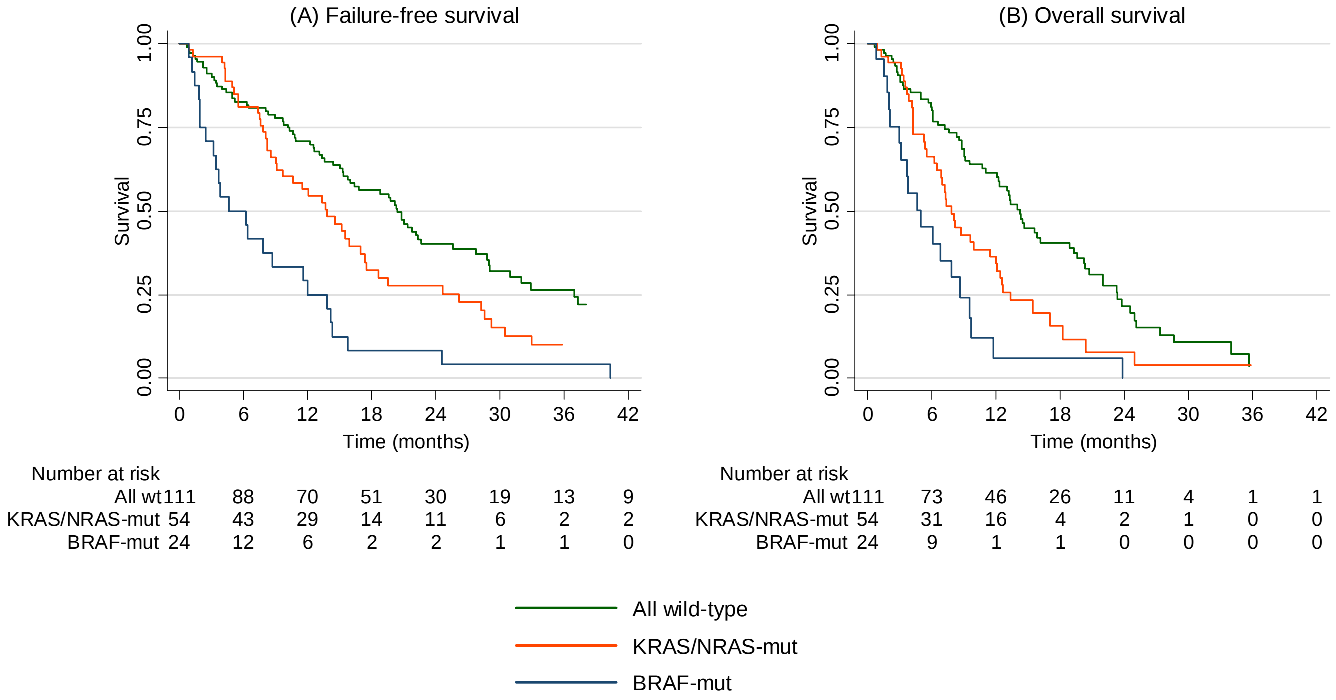

KRAS wild-type patients are those patients who did not have a *KRAS* mutation at codons 12, 13 or 61.

All wild-type patients are those patients who did not have a mutation at any of *KRAS* codons 12, 13 and 61, *BRAF* codon 600 or *NRAS* codons 12, 13 and 61.

Table 1: Description of Patients who Dropped out of Trial Therapy in First 12 weeks

	Intermittent cetuximab		Continuous cetuximab		Total	
	N	%	N	%	N	%
**Total confirmed *KRAS* wild-type**	**78**	**(100%)**	**91**	**(100%)**	**169**	**(100%)**
Progressive Disease in first 12 weeks	1	(1%)	4	(4%)	5	(3%)
Death in first 12weeks	4	(4%)	14	(16%)	18	(11%)
Came off trial in first 12weeks	9	(12%)	7	(8%)	16	(9%)
**Total dropped out in first 12weeks**	**14**	**(18%)**	**25**	**(27%)**	**39**	**(23%)**
**…of whom:**						
BRAF-mutation present *	1	(8%)	8	(33%)	9	(25%)
Aged 75+	1	(7%)	6	(24%)	7	(18%)
WHO PS 2+	2	(14%)	4	(16%)	6	(15%)

* Denominators are slightly different due to missing BRAF data: n=12 (rather that 14) for ICT+IC and n=24 (rather than 25) for ICT+CC

COIN-B Sites, Principal Investigators at each Site and Number of *KRAS* wild-type Patients Randomised at that Site

Cheltenham General & Gloucestershire, Dr Kim Benstead [23]
Essex County Hospital, Dr Bruce Sizer [23]
Royal Surrey County, Prof Gary Middleton [20]
Charing Cross Hospital, Dr Charles Lowdell [20]
Hammersmith Hospital, Dr Harpreet Wasan [16]
Weston Park Hospital, Dr John Wadsley [11]
Darent Valley Hospital, Dr Andrew Gaya [11]
St Mary's Hospital (London), Dr Susan Cleator [9]
Singleton Hospital, Prof. John Wagstaff [8]
Royal Bournemouth Hospital, Dr Tamas Hickish [4]
Peterborough District, Dr Karen McAdam [3]
Churchill hospital, Dr Andrew Weaver [2]
Dorset County Hospital, Dr Richard Osborne [2]
Guys and St Thomas's (London), Dr Andrew Gaya [2]
Hereford County Hospital, Dr Nick Reed [2]
Royal United Hospital, Dr Emma De Winton [2]
Southport and Formby District General, Dr Sun Myint [2]
St Helens Hospital, Dr Ernest Marshall [2]
University Hospital of North Staffordshire, Dr Fawzi Adab [2]
Addenbrooke's Hospital, Dr Hugo Ford [1]
Bank of Cyprus, Dr Demitris Papamichael [1]
Poole Hospital, Dr Tamas Hickish [1]
Warrington & Halton Hospitals, Dr Adrian Moss [1]
Worcestershire Royal Hospital, Dr David Farrugia [1]
Bradford Royal Infirmary, Dr Chris Bradley [0]
Cumberland Infirmary, Dr Jonathan Nicoll [0]
St Georges Hospital (London), Dr Fiona Lofts [0]
Walsall Manor Hospital, Dr Andrew Hartley [0];
Ysbyty Gwynedd, Dr Catherine Bale [0]

In addition to the above-named individuals, we acknowledge the contributions of a large number of clinicians, research nurses, data managers and other clinical and support staff at the participating sites.

Biomarker Analysis – Primer Sequences

Fragment	PCR primers	Pyrosequence primer	Sequence
BRAF codon 600	BRAF 15_biotin_R		[Btn]CCAGACAACTGTTCAAACTGAT
	BRAF 15_F		AAGACCTCACRGTAAAAATAGGTG
		BRAF 15_seq	ATAGGTGAYTTTGGTCTAG
KRAS codons 12-13	KRAS_12&13_F		GGCCTGCTGAAAATGACTGA
	KRAS_12&13_biotin_R		[Btn]AGAATGGTCCTGCACCAGTAATA
		KRAS 12&13_seq	CTTGTGGTAGTTGGAG
KRAS codon 61	KRAS_61_F		TGTTTCTCCCTTCTCAGGATTC
	KRAS_61_biotin_R		[Btn]AAGAAAGCCCTCCCCAGTC
		KRAS_61_seq	GGATATTCTCGACACAGC
NRAS codons 12-13	NRAS_12&13_F		GGTTTCCAACAGGTTCTTGCTGGTG
	NRAS_12&13_biotin_R		[Btn]ACAGGATCAGGTCAGCGGGCT
		NRAS 12&13_seq	TGGTGGTGGTTGGAG
NRAS codon 61	NRAS_61_R		CGCCTGTCCTCATGTATTGGTCT
	NRAS_61_biotin_F		[Btn]TCCACACCCCCAGGATTCTT
		NRAS_61_seq	CTCATGGCACTGTACTCT

Timelines for Prospective KRAS Screening

When KRAS screening was introduced the average time from screening consent to the release of results to allow randomisation was 12 working days (range of 4 to 62 working days).

Permitted Dose Adjustments for the Management of Toxicity (excerpted from the COIN-B protocol)

Allergic/Hypersensitivity reactions - Cetuximab

Please note that a physician must be present in the unit or ward, or immediately available by emergency bleep, during the first infusion of cetuximab and for one hour thereafter. This includes any time when cetuximab is being re-introduced after an interval (either planned OR due to toxicity). For subsequent infusions, a suitably qualified nurse must be present, and a physician within close proximity, or immediately available by emergency bleep. Adrenaline, glucocorticoids and emergency resuscitation equipment must be immediately available.

Severe Grade 3/4 hypersensitivity reactions have occurred in 2.2% of patients treated with cetuximab. One death from angio-oedema has been reported.

Signs include rapid onset of airway obstruction, urticaria and/or hypotension. 80% occur during or within one hour of the first infusion, but reactions can occur with later infusions.

Treatment depends on the Grade or severity of the reaction (see Table 1).

Once the infusion rate has been slowed for an allergic reaction, it should remain at the slower rate for all subsequent infusions.

If the patient has a second allergic/hypersensitivity reaction on the slower infusion rate, the infusion should be stopped and no further cetuximab administered.

If a patient receives a Grade 3 or 4 allergic/hypersensitivity reaction at any time, cetuximab must be discontinued.

If there is any doubt whether a reaction is an allergic/hypersensitivity reaction of Grades 1-4, the CI should be contacted immediately to discuss and Grade the reaction.

Table 1: Toxicity and dose adjustments for FOLFOX plus cetuximab, and during cetuximab monotherapy (ICT+CC)

**CTC Grade Allergic/hypersensitivity reaction**	**Treatment**
Grade 1<br>Transient flushing or rash, drug fever <38ºC	Decrease the cetuximab infusion rate by 50% and monitor closely for any worsening.<br>The total infusion time for cetuximab should not exceed 240 minutes.
Grade 2<br>Rash; flushing; urticaria; dyspnea; drug fever ≥ 38°C	Stop cetuximab infusion.<br>Administer bronchodilators, oxygen etc. as medically indicated.<br>Resume infusion at 50% of previous rate once allergic/hypersensitivity reaction has resolved or decreased to Grade <=1 in severity, and monitor closely for any worsening.
Grade 3 or Grade 4	Stop cetuximab infusion immediately and disconnect infusion tubing from the patient.
Grade 3: Symptomatic bronchospasm, with or without urticaria; parenteral	Administer adrenaline, bronchodilators, antihistamines, glucocorticoids, intravenous fluids,

medication(s) indicated; allergy-related oedema/angioedema; hypotension	vasopressor agents, oxygen etc., as medically indicated.
Grade 4: Anaphylaxis	Patients have to be withdrawn immediately from treatment and must not receive any further cetuximab treatment.

Skin toxicity - cetuximab

The acneiform skin rash associated with cetuximab occurs in over 70% of patients. About 12% have Grade ≥ 3 reactions. On longer-term therapy, paronychia occurs in about 10% of patients and can be painful.

Discussion with a local dermatologist prior to study initiation would be helpful to agree local plans of management and mechanisms for rapid referral in case of severe skin toxicity.

The occurrence of the rash has been correlated with an increased likelihood of response to treatment, though this information needs to be used with caution, as it is not a direct correlation.

The rash usually occurs on the face, upper chest and back with multiple follicles and pustules. The onset is usually within the first 3 weeks of treatment and many cases improve by 12 weeks on treatment.

- Dose modifications are summarised in the algorithm below. For CTC Grade 1 or 2: continue treatment with cetuximab. If a patient experiences Grade 3 skin toxicity (rash effects > 50% of body surface area), cetuximab therapy may be delayed for up to 14 days without changing the dose level. If Grade 3 skin toxicity occurs for a second and third time, cetuximab therapy may again be delayed for up to 14 days with concomitant dose reductions to 200mg/m^2 and then 150mg/m^2. Cetuximab dose reductions are permanent. Patients must discontinue cetuximab if more than 2 consecutive infusions are withheld or Grade 3 skin toxicity occurs for a fourth time despite appropriate dose reduction. If the toxicity resolves to Grade 2 or less by the following treatment period, treatment may be resumed.

- The investigator should also consider concomitant treatment with topical and oral antibiotics. Grade 1 acneiform eruption: consider treatment with topical antibiotics (e.g. topical metronidazole or erythromycin). Systemic antibiotics (e.g. a second generation tetracycline such as doxycycline 100mg po daily) should be considered for Grade 2 acneiform eruption, and are mandatory for Grade ≥3 reactions. The threshold for referral to the dermatology clinic should be planned locally but all patients with Grade ≥3 reactions and probably all with Grade 2 reactions should be referred for advice and management. If pruritus occurs an oral antihistamine is advised. Dry skin often occurs (and may contribute to pruritus) general advice on replacing soap with oil for washing, avoidance of hot water for baths or showers and use of emollient creams are beneficial; topical corticosteroids are not recommended. Fissures may occur in dry skin and topical dressings (e.g. hydrocolloid dressings and as advised by your dermatologist) are helpful.
- Nail toxicities occur in about 10% of patients with cetuximab, characterised by a paronychial inflammation with associated swelling of the lateral skin folds of toes and fingers, especially great toes and thumbs, which may be painful. It may persist for up to 12 weeks after cessation of cetuximab therapy. Dermatological advice should be sought. Use of daily salt baths and local antiseptic / astringent ointments have been found to be helpful. Anti-inflammatory drugs may help to ease the pain.

Figure 1: Treatment adjustments for cetuximab-related skin toxicity:

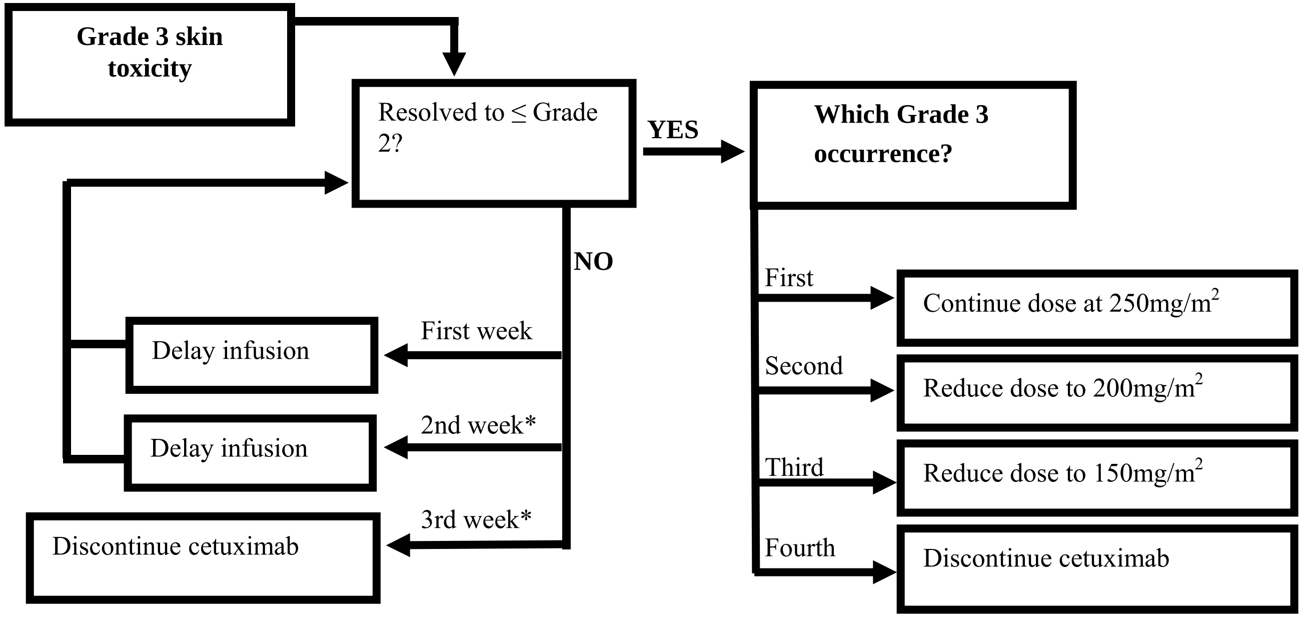

* These refer to the second and third consecutive week of non-resolving Grade 3 skin toxicity.

Hypomagnesemia - cetuximab

Hypomagnesemia has been reported in up to 65% of patients following cetuximab therapy. Patients should have magnesium concentration monitored at baseline, prior to each cycle of chemotherapy and for up to 8 weeks after the last dose of cetuximab, or until magnesium has normalised, whichever is the longer.

Fatigue, malaise, tremor, ataxia, carpopedal spasm, hyperreflexia, confusion, hallucinations, convulsions and arrhythmias may occur.

Hypomagnesemia should be corrected by intravenous supplementation if Grade 3 (<0.4mmol/L) or symptomatic. If lesser degrees of hypomagnesemia are detected, oral supplementation may be considered.

Haematological

Myelotoxicity is similar in frequency as with FOLFOX alone. Leucopenia has been reported and attributed to cetuximab in 8% of patients treated with chemotherapy plus cetuximab.

Check FBC on (or up to 3 working days before) day 1 of each cycle. Delay 1 week if neutrophils <1.5 x 10^9/l or platelets <75 x 10^9/l. Only treat when neutrophils and platelets are above these limits. The lower limit for the day one platelet count for this regimen is due to the possible occurrence of mild thrombocytopenia after a number of cycles of FOLFOX.

If >1 delay, or 1 delay of ≥2 weeks occurs, maintain oxaliplatin and infusional 5FU doses but omit bolus 5FU and continue without bolus 5FU for subsequent doses.

If a further delay(s) for myelotoxicity occurs despite omitting bolus 5FU reduce the oxaliplatin and infusional 5FU doses by 20%.

Further dose reductions may be made, at the discretion of the treating investigator.

Neurotoxicity

Oxaliplatin commonly causes peripheral sensory symptoms, easily distinguishable from 5FU neurotoxicity, which is uncommon, and cerebellar.

Many patients experience transient paraesthesia of hands and feet, and some experience dysaesthesia in the throat. These symptoms are precipitated by cold and last from a few hours to a few days after each oxaliplatin administration. They do not require treatment or dose reduction.

If symptoms persist for 14 days (i.e. until the next cycle is due), and are associated with significant discomfort or loss of function (e.g. dropping objects), omit oxaliplatin from the regimen and continue with MdG plus cetuximab until symptoms resolve to Grade 1, then reintroduce oxaliplatin with the dose reduced to 75mg/m^2.

Renal function

Oxaliplatin, like carboplatin, is not nephrotoxic but is renally cleared.

Before starting oxaliplatin (or if patient switched to capecitabine), ensure patient fulfils eligibility for renal function.

Check serum creatinine at each cycle. If this rises >25%, re-check EDTA clearance or 24-hour urinary creatinine, and adjust oxaliplatin and 5FU doses according to the table in Appendix VI of protocol.

If GFR drops to below 30ml/min, omit oxaliplatin and reduce 5FU by 25% until recovery.

There is little experience of administering cetuximab in patients with renal insufficiency. Physicians should exercise caution and consider a dose reduction. No specific guidelines are available, however investigators may discuss dose-reduction with the CI for the trial.

Hepatobiliary function

Oxaliplatin is not principally cleared by the liver, but there is evidence of delayed clearance in patients with marked hepatic dysfunction. For this reason oxaliplatin and 5FU should be reduced by 50% in patients with serum bilirubin >3 x ULN.

Bilirubin ≤1.25 x ULN is required for study entry. If bilirubin rises above this limit during treatment, discuss with investigator as this may indicate disease progression.

Transaminase (either AST or ALT) ≤ 2.5 x ULN is required for study entry. An isolated rise in transaminase to 2.5 x ULN during treatment is likely to be treatment-related, and both bolus and infusional 5FU treatment should be interrupted until recovery.

There is little experience of administering cetuximab to patients with hepatic insufficiency. Physicians should exercise caution and consider a dose reduction. No specific guidelines are available, however investigators may discuss dose-reduction with the CI for the trial.

Respiratory

As with other platinum drugs, rare cases of acute interstitial lung disease or lung fibrosis have been reported with oxaliplatin. In the case of unexplained respiratory symptoms or signs, oxaliplatin should be discontinued until further pulmonary investigations exclude an interstitial lung disease.

Interstitial pneumonitis

A syndrome of severe acute interstitial pneumonitis has been reported recently with another EGFR targeted therapy, gefitinib.

Should a patient develop severe dyspnoea and/or hypoxia, seek urgent assistance from a chest physician. A high resolution CT scan of thorax, bronchoscopy plus transbronchial biopsies for pathology and relevant cultures should be considered.

Cetuximab should be withheld until complete resolution of the toxicity.

Stomatitis

Routine mouthcare (e.g. Corsadyl, nystatin) is recommended.

If mouth ulcers occur despite this, reduce the 5FU doses (bolus and infusion) by 20% and continue at the lower dose for subsequent cycles unless further toxicity occurs.

If further toxicity occurs reduce 5FU (bolus and infusion) and oxaliplatin doses by a further 20%.

Stomatitis occurs in about 10% of patients treated with cetuximab. It has not been considered an indication for dose reduction in previous trials. However, if stomatitis continues despite 5FU and oxaliplatin dose reductions, cetuximab dose reduction to 200mg/m^2 should be considered.

Diarrhoea

Diarrhoea Grade 3/4 has been reported in 6% of patients treated with cetuximab plus chemotherapy and 4 serious adverse events due to diarrhoea were reported in the phase 2 trial of cetuximab plus FOLFOX.

For diarrhoea occurring between cycles, treat symptomatically initially: loperamide 2-4 mg qds and/or codeine phosphate 30-60 mg qds as required.

If diarrhoea has not resolved by the time the next cycle is due, delay 1 week.

If diarrhoea is a problem despite symptomatic treatment, or if more than one delay is required, reduce the oxaliplatin and 5FU (bolus and infusion) doses by 20% and continue at the lower dose for subsequent cycles unless further toxicity occurs.

If further toxicity occurs reduce 5FU (bolus and infusion) and oxaliplatin doses by a further 20%.

If diarrhoea persists despite this dose reduction, cetuximab dose reduction to 200mg/m^2 should be considered.

Hand-foot syndrome (HFS)

Treat symptomatically. Pyridoxine 50 mg tds by mouth or topical corticosteroids may help.

If HFS is still a problem, reduce the 5FU doses (bolus and infusion) by 20% for subsequent cycles.

If further toxicity occurs reduce 5FU (bolus and infusion) and oxaliplatin doses by a further 20%.

DPD deficiency; cardiotoxicity

DPD is the initial and rate-limiting enzyme for 5-FU breakdown. DPD deficiency is an inherited (pharmacogenetic) disorder in which individuals with absent or significantly decreased DPD activity develop life-threatening toxicity following exposure to 5-FU (in either IV or oral form). Reduced drug clearance results in markedly prolonged exposure to 5-FU so that administration of standard doses of 5-FU results in altered 5-FU pharmacokinetics and severe toxicity including mucositis, granulocytopenia, neuropathy and death.

The onset of toxicity usually occurs twice as fast in patients with low DPD activity as compared with patients with a normal DPD activity. Approximately 3-5% of the population has low DPD activity and 0.1% have absent activity.

We recommend that (i) patients with a personal or family history suggestive of DPD deficiency should not be enrolled onto COIN-B, and (ii) those who experience grade 3/4 neutropaenia and grade 3/4 mucositis after cycle 1 should be considered as potentially having DPD deficiency. If DPD deficiency is suspected, patients should only continue on trial after full recovery but without the further use of a fluoropyrimidine.

5FU may provoke angina attacks or even MI in patients with ischaemic heart disease. Continued treatment with upgraded antianginal medication and reduced 5FU dose may be considered.

Allergic reactions to oxaliplatin

The occasional patient (approx. 0.5%) develops acute hypersensitivity to oxaliplatin, usually after more than 6 cycles have been administered. During drug administration, the patient may develop rash, fever, swollen mouth or tongue, hypo- or hypertension and other signs/symptoms of hypersensitivity. This rarely develops to full-blown anaphylaxis, even with repeated treatment.

If acute hypersensitivity occurs, discontinue the infusion and treat with IV corticosteroid and antihistamine.

After full recovery, the patient may continue with cetuximab, FA and 5FU.

At the investigator's discretion, the patient may be rechallenged with oxaliplatin at the next cycle. In this case, premedication is recommended as follows:

- Dexamethasone 4mg po 6 hourly starting 24 hours pre-treatment, + 8mg IV 30 minutes pre-dose Chlorphenamine 10mg (or equivalent) + ranitidine 50mg (or equivalent) IV 30 minutes pre-dose.

Continue dexamethasone, chlorphenamine and ranitidine for 24-48 hours after treatment with oxaliplatin.
